# Mechanical interplay between adipose tissues and disease progression

**DOI:** 10.1002/btm2.70065

**Published:** 2025-10-02

**Authors:** Hangyu Zhou, Danni Zhou, Miaoben Wu, Yuye Huang, Enxing Yu, Jianing Xie, Yangjian Wang, Shuqin Chen, Qinghua Song, Kailei Xu, Peng Wei

**Affiliations:** ^1^ Department of Hand and Microsurgery, People's Hospital of Zhenhai District (Ningbo No.7 Hospital), Ningbo, Zhejiang 315202, China; ^2^ Department of Plastic Surgery The Affiliated People's Hospital of Ningbo University Ningbo Zhejiang China; ^3^ Health Science Center Ningbo University Ningbo Zhejiang China; ^4^ Department of Plastic and Reconstructive Surgery The First Affiliated Hospital of Ningbo University Ningbo Zhejiang China; ^5^ Department of Plastic and Reconstructive Surgery, Guang Dong Second Provincial People's Hospital The Affiliated Guangdong Second Provincial General Hospital of Jinan University Guangzhou Guangdong China; ^6^ Center for Medical and Engineering Innovation, Central Laboratory The First Affiliated Hospital of Ningbo University Ningbo Zhejiang China; ^7^ Hangzhou No. 14 High School Hangzhou Zhejiang China; ^8^ Department of Endocrinology and Metabolism The First Affiliated Hospital of Ningbo University Ningbo Zhejiang China

**Keywords:** adipocytes, bone defects, cancer, cardiac diseases, stiffness

## Abstract

Over the past two decades, an increasing body of evidence has underscored the significant role of the mechanical properties of biological tissues in maintaining tissue functions and regulating cellular changes, such as proliferation, migration, and differentiation. Throughout disease progression, such as in cancers, bone defects, and cardiac conditions, the mechanical microenvironment of tissues can undergo dramatic changes, exerting profound effects on disease development. Adipose tissues are inherently mechanosensitive and mechanoresponsive, continually exposed to various mechanical stresses in daily life. The hypertrophy and accumulation of adipocytes can lead to obesity, a condition strongly associated with numerous health risks, like diabetes and cancers. In this review, we aim to elucidate the reciprocal mechanical interaction between adipose tissues and disease progression, encompassing cancers, bone defects, and cardiac pathologies. The existing literature suggests that alterations in the mechanical microenvironment during disease advancement may impede adipogenic differentiation, induce adipocyte dedifferentiation, and escalate the secretion of inflammatory cytokines. Conversely, dysregulation of adipose tissues can result in the deposition of extracellular matrix components, stiffening the microenvironment and fostering disease progression in a cyclical fashion. Therefore, in future treatments of related diseases, a combined approach integrating mechanotherapeutics and obesity management holds promise for achieving the desired enhanced therapeutic outcomes.

**Translational Impact Statement**

Over recent decades, a growing body of evidence has shown that the mechanical properties of biological tissues critically regulate the progression of diverse diseases, including cancer, skeletal defects, and cardiovascular disorders, and that adipose tissue, as a mechanosensitive and mechanoresponsive organ, continuously encounters compound mechanical stresses that influence its function and disease‐related signaling. However, the bidirectional interactions between altered adipose mechanics and disease progression remain poorly synthesized. In this review, we integrate current findings on how changes in tissue mechanics drive disease, delineate the promotional roles of adipose tissue in malignancy, bone pathology, and myocardial dysfunction, highlight the reciprocal mechanical dialog between adipose remodeling and disease states, and evaluate the therapeutic potential of combining mechanotherapeutic interventions with obesity‐targeted treatments.

## 1 INTRODUCTION

Human tissues exhibit a wide range of mechanical properties, from soft fat tissue in the Pascal (Pa) range^1^ to rigid bone at the Gigapascal (GPa) scale,^2^ playing a critical role in maintaining tissue functions and regulating cellular proliferation, migration, and differentiation. For instance, the osteogenic or adipogenic differentiation of mesenchymal stem cells (MSC) depends on the stiffness of the cell culture substrate.^3^ During the progression of disease, such as cancers, bone defects, and cardiac fibrosis, the tissue mechanical microenvironment can also change dramatically and promote disease development.

Obesity is one of the most significant global health issues, with an estimated >603.7 million obese adults according to the Global Burden of Disease Obesity Collaborators.^4^ Obesity is strongly correlated with the risk of numerous other diseases, such as heart disease,^5^ high blood pressure,^6^ liver disease,^7^ and certain cancers.^8^, ^9^ Adipose tissues are continuously subject to compound mechanical stimuli, including tensile, compressive, and shear stresses.^10^ Those mechanical cues could influence the differentiation of adipose‐derived stem cells (ADSCs) in the bone microenvironment.^11^ Relatively higher stiffness was also found to inhibit the adipogenesis of preadipocytes.^12^ Mature adipocytes have demonstrated dedifferentiation and regained stemness properties under compression, mediated by mechanically activated Wnt/β‐catenin signaling.^13^

This review focuses on the reciprocally mechanical interactions between adipose tissue and disease progression. Specifically, for people with diabetes, adipocyte hypertrophy and accumulation contribute to mechanical stress inside the adipose tissues and also exert compressive forces on the surrounding microenvironment. Conversely, changes in the mechanical properties of the microenvironment during disease progression can provide physical cues that influence adipocyte behavior.

## 2 INFLUENCE OF ADIPOSE TISSUE ON DISEASE PROGRESSION

### 2.1 Adipose tissues

**FIGURE 1:**
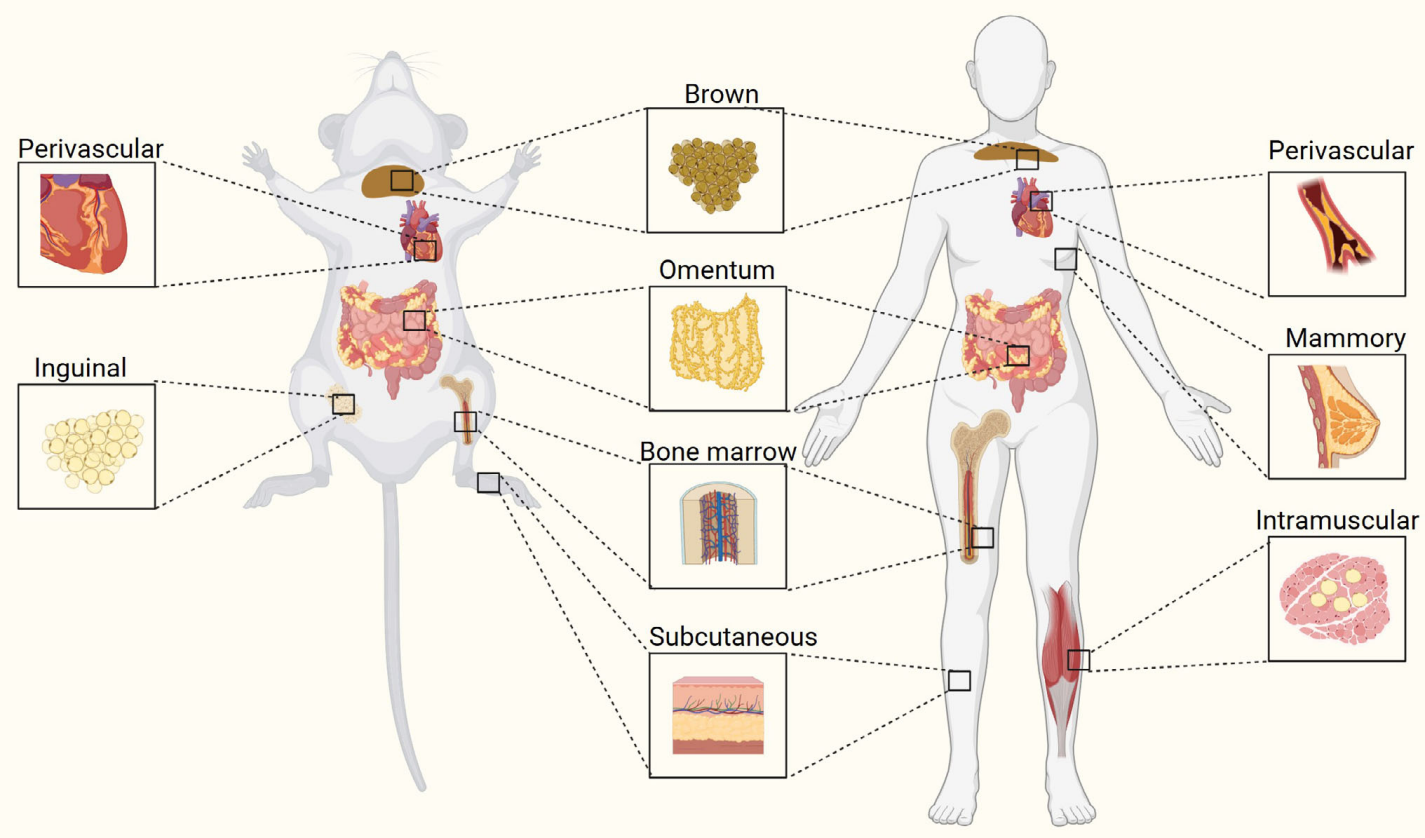
Adipose tissues exist in almost all tissues and organs, constituting 9% to 18% of body weight in lean males and 14%–28% in lean females. Created with BioRender.com.

**FIGURE 2:**
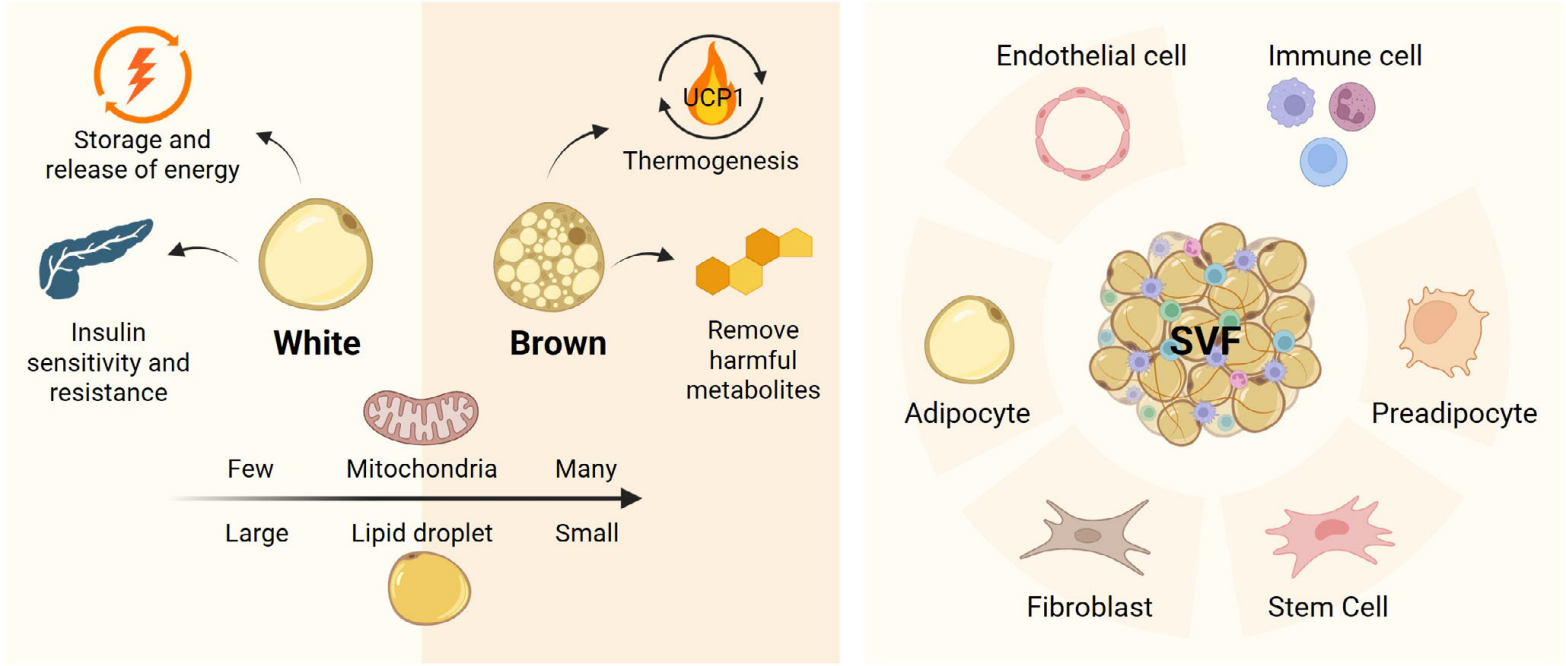
Most mammal adipose tissue cells include white, beige, and brown adipocytes, as well as stromal vascular fraction (SVF) cells. White adipocytes are adapted to storing and releasing lipids, whereas beige and brown adipocytes are specialized thermogenic cells that can utilize dietary energy by generating heat. SVF cells are nonadipocytes in adipose tissues. Created with BioRender.com. UCP1, uncoupling protein 1.

Adipose tissue, also known as fat or fatty tissue, comprises of a different type of connective tissue mainly composed of adipocytes (Figure 1). These are multi‐located tissues in the three major anatomical—subcutaneous, dermal, and visceral—depots^14^ (Figure 2) and can be classified as white adipose tissue (WAT), brown and beige adipose tissue, and stromal vascular fraction (SVF).

The predominant type of adipose tissue is WAT, present in nearly all regions of the body, which are categorized based on their anatomical positioning as either subcutaneous or visceral.^15^ White adipocytes are characterized by the presence of a solitary, substantial lipid droplet that occupies a significant portion of the cell, accompanied by relatively small mitochondria.^16^ The primary role of white adipocytes is to store and release energy in response to fluctuations in overall energy levels within the body. These activities occur across various timeframes, with lipolysis (the release of fatty acids) and lipogenesis (the uptake or synthesis of fatty acids) operating on a short‐term basis.^17^ WAT is also an essential endocrine organ that secretes adipokines to regulate whole‐body metabolism, promoting insulin sensitivity, resistance, and inflammation.^18^ In obesity, hypertrophic adipocytes and adipose tissue‐resident immune cells facilitate a chronic, proinflammatory profile with an altered secretion of adipokines and lipokines, thereby increasing the risk of many pathologies, including cardiometabolic disease,^5^ hypertension,^6^ and several cancers.^8^, ^9^ Preclinical and clinical studies have indicated that modulating the signaling of specific adipokines or lipokines can be a viable approach for treating or preventing the onset of cardiometabolic diseases.

Brown and beige adipocytes, occupying a small proportion of total adipose tissues, are mitochondria‐enriched cells capable of dissipating energy in the form of heat. They can be distinguished by several key features, including the presence of multilocular lipid droplets, a high density of mitochondria, and the expression of uncoupling protein 1 (UCP1). These two kinds of adipocytes have a significant metabolic effect because they possess the ability to participate in thermogenesis, where UCP1 functions by disrupting the proton gradient in the inner mitochondrial membrane, effectively uncoupling nutrient catabolism from adenosine triphosphate (ATP) synthesis and releasing potential energy in the form of heat.^19^, ^20^ Apart from reducing weight gain through increased energy expenditure, brown adipose tissues (BATs) could also secrete molecules, known as batokines, that influence the physiological functions of various organ systems and signal to a variety of cell types.^21^, ^22^ For instance, BAT releases fibroblast growth factor 21 (FGF21) into the bloodstream to regulate target organs.^23^, ^24^ In addition, increased vascular endothelial growth factor A (VEGFA) expression by activated BAT promotes autocrine and endocrine signaling, exerting a key function in energy metabolism.^25^ In diet‐induced obesity models, a reduction in VEGFA gene expression leads to progressive BAT dysfunction, characterized by decreased vascularity, increased whitening, and a reduced number of brown adipocytes.

SVF as one of the most important components in adipose tissues^26^ comprises a diverse cell population, including endothelial cells, ADSC, fibroblasts, pericytes, preadipocytes, and immune cells.^27^ Among these, the most significant attention has been directed toward understanding the characteristics and functions of ADSC as they have similar morphological features, immune phenotype, colony frequency, and differentiation capacity as bone marrow stem cells (BMSC).^28^ In addition, ADSC displays a greater capacity for proliferation and more effective angio‐inductive capabilities compared to BMSC.^29^ However, the proportion of ADSC in SVF is at least 500 times higher than BMSC found in the bone marrow, which suggests that ADSC can be a more suitable and abundant source for cell therapy and tissue engineering.^30^ Endothelial cells in SVF can autonomously form a hierarchical, branched, and functional vascular network, essential contributors to neovascularization, hemostasis, and immune responses.^31^

### 2.2 Roles of adipose tissues in disease progression

#### 2.2.1 Cancer

**FIGURE 3:**
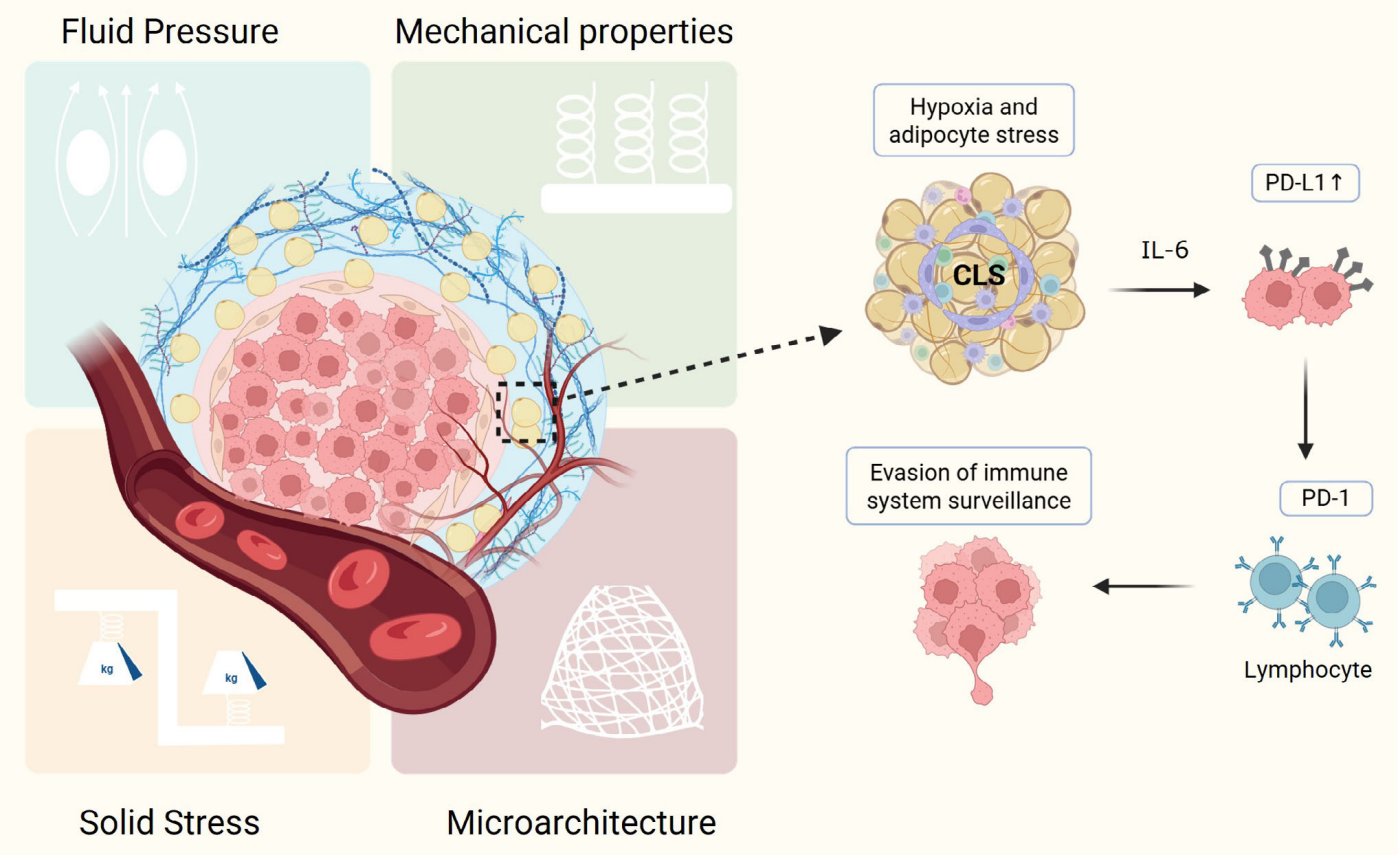
The four primary mechanical stimuli within human organs are deregulated in the tumor microenvironment, including (1) increased solid stress, (2) increased interstitial fluid pressure, (3) altered extracellular matrix (ECM) mechanical properties, and (4) altered ECM microarchitecture. Created with BioRender.com. IL‐6, interleukin 6.

Research has established a positive correlation between obesity and cancer occurrence (Table 1): clinical evidence has increasingly indicated that individuals with obesity face an increased risk of developing various cancers (Figure 3). Adipocytes surrounding tumors constitute a significant component of the tumor stroma, which is a critical source of energy required for tumor development. More specifically, adipocytes can secrete various adipokines to promote tumor cell metastasis, and the consumption of saturated fats may potentially affect cancer recurrence, progression, and mortality.^40^ CCL7, an adipokine secreted by adipocytes, can bind to the CCR3 receptor on prostate tumor cells, initiating cancer cell migration and dissemination.^32^ Expanding adipose tissue triggers tissue inflammation, a potential link between obesity and cancer, causing hypoxia and adipocyte stress.^41^ This leads to increased Monocyte Chemoattractant Protein‐1 (MCP‐1) and cytokine production, attracting and proliferating macrophages and forming crown‐like structures (CLS) indicative of inflammation.^42^ In breast cancer, inflammation initiated by CLS can activate nuclear factor‐κB and promote tumor development.^34^ WAT inflammation during mastectomy for early‐stage breast cancer has been linked to a shorter distant recurrence‐free survival in metastatic cases.^35^ In early‐stage tongue cancer, CLS in tongue fat was also connected to reduced disease‐specific and overall survival.^36^ In addition, adipocytes can also secrete interleukin (IL)‐6 to induce programmed death ligand 1 (PD‐L1) overexpression in cancer cells, which can thus bind to the programmed cell death protein 1 (PD‐1) receptor on the surface of immune cells and escape immune surveillance during metastasis.^33^

**TABLE 1:** Influence of adipose tissues in disease progression.

Adipose tissues		Diseases	Cytokines/ECM	Key research points	References
	Periprostatic adipose tissue	Prostate cancer	CCL7	Cancer cell migration and dissemination was initiated	Laurent et al.^32^
			IL‐6	PD‐L1 overexpression was induced in cancer cells that helped them escape from immune system surveillance during metastasis	Xu et al.^33^
	Breast white adipose tissue	Breast cancer	Inflammatory cytokines	Activate crown‐like structures (CLS) formed from macrophages and reduced overall survival	Morris et al.,^34^ Iyengar et al.^35^
	Tongue white adipose tissue	Oral cancer	Inflammatory cytokines	Correlation with disease‐specific survival	Iyengar et al.^36^
	BMAT	Osteoporosis	Peroxisome proliferator‐activated receptor gamma (PPARγ)	PPARγ was upregulated in systemic obesity and reduced differentiation of progenitors to osteoblasts	Duque et al.^37^
	BMAT	Osteoarthritis	Mechanical loading	Increased adipose tissues led to weight gain, resulting in abnormal loading on the joints.	Zapata‐Linares et al.^38^
			Interleukin 1 Beta (IL‐1β), TNF‐α	Cartilage breakdown inhibited chondrocyte proliferation and caused the degradation of essential matrix components	Wang et al.^39^

Abbreviations: BMAT, bone marrow adipose tissue; ECM, extracellular matrix; IL‐6, interleukin 6; TNF‐α, tumor necrosis factors‐α.

#### 2.2.2 Bone diseases

Adipose tissues in the bone microenvironment mainly exist in the bone marrow, named as bone marrow adipose tissue (BMAT), which is found within medullary canals of long bones such as the tibia, femur, and humerus, and in flat bones such as the sternum and iliac crest.^43^ BMAT is widely recognized for its unique characteristics, including distinct development, molecular makeup, and regulatory mechanisms, setting it apart from other adipose tissues. Increases in BMAT volume have been associated with various physiological and pathological conditions, such as aging,^44^ type 2 diabetes mellitus (T2DM), and osteoporosis^45^ (Table 1).

Osteoporosis results from an imbalance between the functioning of osteoclasts and osteoblasts.^46^ Adipocytes and osteoblasts originate from a shared precursor known as pluripotent BMSC, which possesses an equal potential for evolving into adipocytes or osteoblasts and other cell types such as endothelial cells, fibroblasts, and chondrocytes.^47^ Currently, the prevailing hypothesis posits that the inability of BMSC to differentiate into osteoblasts results in an increased propensity for differentiation into adipocytes. This is because in osteoporosis, there exists an inverse relationship between fat production in the bone marrow and bone formation, as reduced adipogenesis has been observed in individuals with high bone mass.^48^ The relationship between osteoarthritis (OA) and obesity has also been frequently attributed to the effects of mechanical overload due to excess weight. Clinical studies have consistently reported positive associations between body mass index (BMI) and the development and advancement of knee OA.^49^ Adipose tissues can affect OA through metabolic pathways,^50^ in which they can disrupt lipid profiles and release adipokines, contributing to the development of the condition. For example, visceral adipose tissues can release inflammatory cytokines such as IL‐1β^51^, ^52^ and tumor necrosis factor (TNF)‐α,^53^ examples of cytokines well documented for their active involvement in the pathophysiology of OA.

#### 2.2.3 Cardiac diseases

Increasing evidence suggests that obesity is a significant risk factor for heart disease.^54^ Adipose tissue expansion and dysfunction have been shown to lead to cardiovascular disease (CVD) through a series of interrelated direct and indirect mechanisms. Based on their location, adipose tissues in the heart can be classified as epicardial adipose tissue (EAT) and perivascular adipose tissue (PVAT). EAT refers to adipose tissues between the myocardium and the visceral pericardium, extending toward the apex and along the coronary arteries.^55^ Compared to other fat depots, EAT features a higher density of adipocytes per gram, smaller‐sized adipocytes,^56^ and disparities in protein content and fatty acid composition. On the other hand, PVAT refers to fat surrounding blood vessels, enveloping them without a fascial layer to separate them from the vascular wall. The classification of PVAT into white or brown adipose tissue remains a subject of debate. Despite the higher expression levels of certain brown adipocyte markers,^57^ UCP1 levels are ~1000‐fold lower than in brown adipocytes.^57^ Other studies have suggested a predominant white adipocyte‐like phenotype in PVAT.^58^ Apparently, the physiology and function of PVAT may exhibit multifaceted variations across different blood vessels. PVAT is a pivotal regulator of vascular function due to its proximity to the vascular wall, exerting a spectrum of direct paracrine effects.^59^ Under healthy conditions, adipose tissues are vital in controlling vascular tone by releasing molecules with vasorelaxant properties. However, in conditions such as obesity and insulin resistance, the production of adipokines is diminished, potentially contributing to vasomotor dysfunction associated with obesity.^60^

## 3 MECHANOSIGNALING IN TISSUE PHYSIOLOGY AND DISEASE PROGRESSION

### 3.1 Physical traits

**FIGURE 4:**
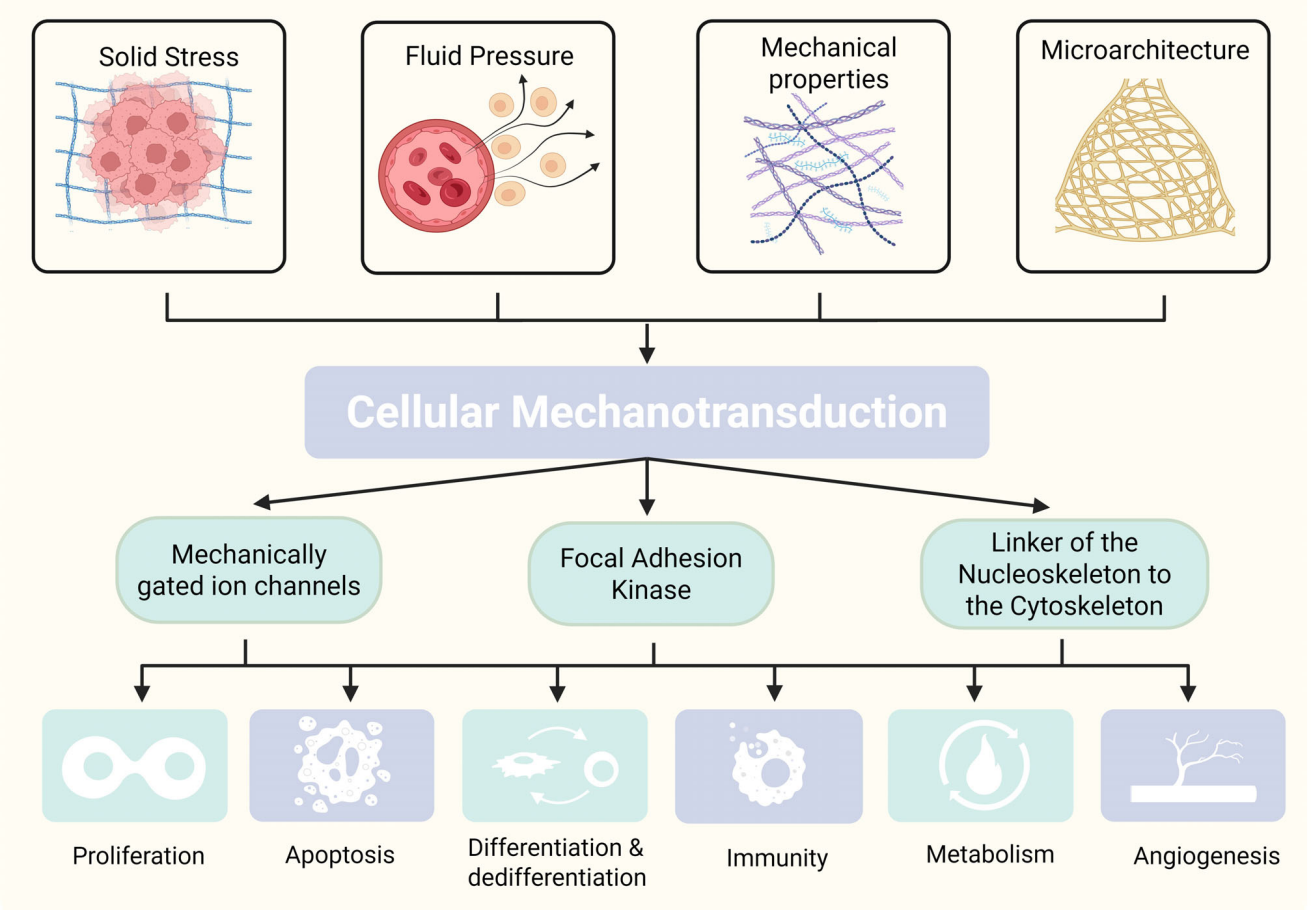
The four categories of mechanical cues in human organs and tissues are solid stress, interstitial fluid pressure, mechanical properties, and microarchitecture, which can be transduced via mechanotransduction and influence cell behaviors. Created with BioRender.com.

Changes in the mechanical properties can play a critical role in maintaining the function of tissues and organs,^61^ controlling pathological processes,^62^ manipulating immune responses,^63^ and influencing cell fate decisions.^64^ Mechanical cues in human organs and tissues can be divided into four categories: solid stress, interstitial fluid pressure (IFP), ECM mechanical properties, and ECM microarchitecture^65^ (Figure 4).

#### 3.1.1 Solid stress

Solid stress, also known as residual stress, refers to the latent mechanical stress stored within and transmitted by the solid and elastic components of the ECM and cells. It is commonly generated in highly proliferative regions of organs and tissues, such as the normal intestinal epithelium, where cell layers turnover every few days, or uncontrolled cancer cell proliferation that can add volume that compresses or stretches ECM components inside or outside the tumor microenvironment (TME).^66^ Adipocytes can also experience increased solid stress caused by compressive forces due to increased cell accumulation and size during obesity, which can initiate a perpetual inflammatory cycle and the pathophysiological signaling pathway.^67^ Cell migration or infiltration with the least resistant path in ECM components or release of proteases, such as those of the matrix metalloproteinase (MMP) family, can result in high tension, which is also solid stress.^68^ In normal tissues, tension is regulated by cell–cell or cell‐matrix interaction, a critical regulator during embryo and organ morphogenesis, whereas abnormal tension can influence cell fate decisions, tissue folding, and the formation of multiple organs. ECM swelling is commonly observed in the TME. Due to its high net negative charge, hyaluronan (HA) overexpression can form gel‐like regions within the tissue, and the resulting repulsive electrostatic forces cause chemical expansion, leading to increased swelling and solid stress.^69^

#### 3.1.2 Interstitial fluid pressure

IFP and solid stress are independent mechanical stresses with distinct origins and consequences.^70^ IFP includes hydrostatic pressure, osmotic pressure, and shear force.^71^ Hydrostatic pressure is vital both in vivo and in vitro for regulating cell differentiation, proliferation, and apoptosis,^72^ which can arise from disease‐related hypertension^73^ or tumor progression.^74^ Osmoregulation is an essential process at the cellular level, enabling cells to react to alterations in ion concentrations in the surrounding microenvironment. The disruption of external osmotic pressure in return can lead to alterations in cell volume, increased molecular crowding within the cell due to water loss, and modifications in cell stiffness, ultimately influencing the osteogenic or adipogenic differentiation of MSC.^75^ Shear stress is the quantification of the frictional force exerted by a fluid on the object positioned in the flow, just like the force of flowing water on the channel bed. The most commonly observed shear stress in the human body is generated by blood flow, which is constantly exerted on blood vessels and stimulates endothelial cells.^71^ Shear stress dysregulation in the TME is commonly observed due to neoangiogenesis, which triggers cancer‐associated fibroblast (CAF) activation,^76^ stimulates MMP activity, and promotes cell motility.^77^ The adipogenic differentiation process of preadipocytes is also regulated by shear stress, where high shear stress can suppress adipocyte maturation.^78^

#### 3.1.3 ECM mechanical properties

The mechanical properties and microarchitecture of the ECM are also critical biomechanical cues that can regulate cell behaviors.^79^ These two mainly depend on the cellular sensing of the surrounding microenvironment, which can be recognized as nonliving biomechanical cues, unlike solid stress and fluid pressure.^65^

Stiffness, also known as elasticity, is one of the most widely investigated among matrix mechanical properties. Cell behaviors can be strongly influenced by ECM stiffness. For instance, the adipogenic differentiation of MSCs can be activated in the ECM with relatively smaller stiffness.^80^ Endothelial cells are also sensitive to changes in ECM stiffness, resulting in abnormal angiogenesis and vessel permeability.^81^ Elevated tissue stiffness is the most palpable and widely acknowledged mechanical anomaly observed in tumors, such as breast,^82^ prostate,^83^ and pancreatic cancer,^84^ and is also a diagnostic or prognostic marker.

Apart from stiffness, there is growing recognition of the significance of other mechanical properties, including nonlinear elasticity, viscoelasticity, and plasticity. Nonlinear elasticity can stiffen with growing strain, preventing tissue damage under severe stresses or large deformations.^85^ Viscoelastic properties are characterized by a time‐dependent mechanical response and the release of some energy absorbed during deformation.^86^ Obese fat tissues are more viscoelastic than lean tissues, which can be caused by the adipogenic differentiation process of preadipocytes that alter cells' viscoelastic properties.^87^ The stress relaxation can also influence chondrocyte growth and is a key design parameter for cartilage tissue engineering.^88^ Plasticity is another well‐known property in the ECM, indicating an irreversible deformation after mechanical loading is removed.^89^ Plasticity can independently regulate MSC spreading through ECM adhesion and remodeling genes.^90^

#### 3.1.4 ECM microarchitecture

ECM microarchitectures, such as pore size, porosity, and alignment, are important for optimizing tissue stability, efficiency, and function. Pore size and porosity are vital factors that regulate biomolecular diffusion and metabolism exchange, which can influence cell viability and proliferation in 3D cell culture.^91^ A recent study has concluded that microporous/nanoporous struts developed by 3D printing could influence MSC adhesion, morphology, and differentiation to chondrogenic and osteogenic lineages.^92^ The topography of cell culture substrate, such as continuous, discontinuous, and random roughness, significantly influenced stem cell adhesion, proliferation, and differentiation.^93^ During cancer progression, the matrix microarchitecture also plays a central role in metastasis and therapy, with collagen organization being a prognostic biomarker.^94^ The hierarchical helical structures in muscle and tendon can also prevent cells from damage under these circumstances.^95^

### 3.2 Biomaterial substrates used to analyze physical traits

Traditionally, tissue culture plates made of glass or plastic were used for cell cultures and biological analyses, whereas their material properties are difficult to modify, limiting the investigation of different physical traits on cell behavior. As the development of biomaterials progresses, more and more materials with adjustable mechanical properties have been used to culture cells, to better mimic the ECM chemical compositions and physical properties. Polyethylene glycol (PEG) is a commonly used synthetic polymer for medical applications due to its good biocompatibility and readily adjustable mechanical properties. With increasing PEG concentration (in the range 10%–50% PEG), the moduli could be improved from 2 to 1300 kPa, making it a widely used medium for systematically exploring cell response to specific alterations in substrate stiffness, like adipocytes,^13^ preadipocytes,^12^ fibroblast,^96^ and tenocyte.^97^ Similar synthetic polymers also include polydimethylsiloxane (PDMS), which could be used to analyze the effect of cyclic stretch on cell behaviors due to its good elasticity.^98^

Compared with synthetic polymers, natural polymers can better mimic the ECM compositions of the tissue microenvironment. Agarose is a natural polysaccharide polymer that is commonly used for cell culture due to its excellent biocompatibility and thermo‐reversible gelation behavior. The mechanical property of agarose can be adjusted from 1 to 500 kPa, which can be further combined with external physical stimulations to evaluate the cell behavior. ADSCs embedded in agarose hydrogel can be exposed to hydrostatic pressure to more closely simulate the in vivo condition of the bone marrow microenvironment.^11^ Collagen is the most abundant protein in the human body and a key structural component of various tissues, which has found widespread application in in vitro cell cultures. Meanwhile, due to the relatively low mechanical properties, it has commonly been commonly coated on a more rigid substrate for the investigation of cell mechanotransduction.^99^ Gelatin is a natural form of hydrolyzed collagen, retaining its high biocompatibility and cell adhesion properties. Native gelatin can also form hydrogel only by thermo‐crosslinking like collagen, while it has relatively stronger mechanical properties. Hybrid scaffolds made from gelatin and chitosan could withstand 5% compressive strain in the analysis of ADSC responses.^100^ The methylation modification of gelatin yields a more stable hydrogel, gelatin‐methacryloyl (GelMA), which has been extensively used for cell culture and cell‐laden bioprinting due to its adjustable mechanical properties, rapid gelation, and inherent Arginine‐Glycine‐Aspartic Acid (RGD) sequences. GelMA‐based hybrid hydrogels with various stiffness have been used to analyze the reciprocal interaction between preadipocytes and cancer cells.^12^ Recently, decellularized extracellular matrix (dECM) has gained significant attention from researchers due to its ability to retain the bioactive components and mechanical properties of the ECM, effectively minimizing immune rejection and achieving good tolerance by heterologous hosts.^99^ Decellularized matrices derived from obese ADSC^101^ or preadipocytes^102^ had different stiffness compared with lean ones, which could be used for cell cultures to analyze the influence on cell behavior.

## 4 MECHANICALLY RECIPROCAL INTERACTION BETWEEN ADIPOSE TISSUES AND DISEASE PROGRESSION

### 4.1 Cancer

**FIGURE 5:**
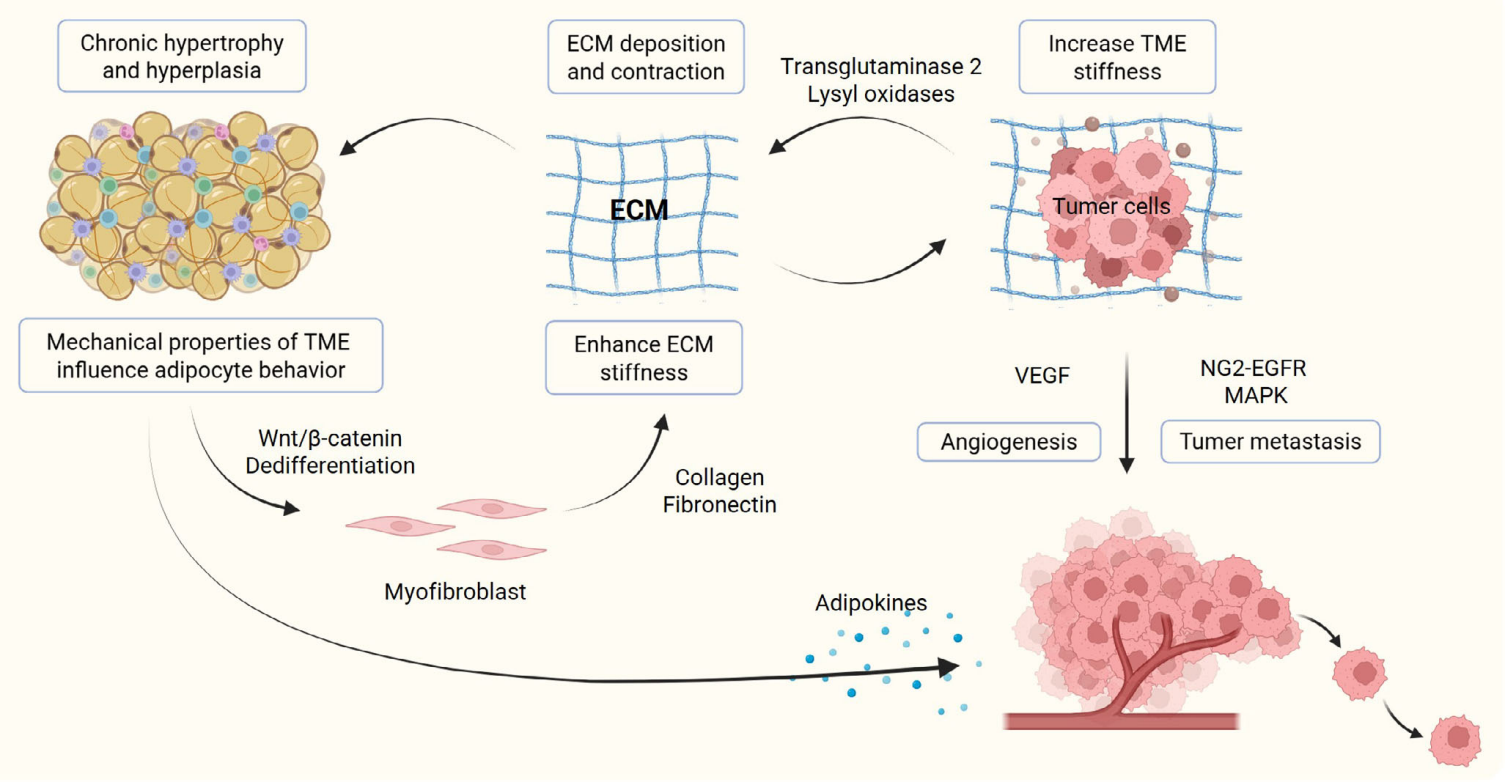
For cancers, cells within adipose tissues establish a detrimental cycle. They contribute to the deposition of extracellular matrix (ECM) components and adipokines, elevating the stiffness of the tumor microenvironment (TME) and fostering the progression, metastasis, and drug resistance of cancer cells. Simultaneously, this process can hinder adipogenic differentiation, trigger adipocyte dedifferentiation, and amplify adipokine secretion, further fueling cancer development. Created with BioRender.com. VEGF, vascular endothelial growth factor; MAPK, mitogen‐activated protein kinase.

Adipose tissues in the TME have a reciprocal interaction with mechanical cues (Table 2), where adipose‐related cells can deposit ECM components and various cytokines to modify the TME mechanical properties. Deregulated physical traits can also change cell behavior, including dedifferentiation and adipokine secretion (Figure 5).

**TABLE 2:** Mechanical cues and adipose tissues in the musculoskeletal systems.

Adipose tissues	Diseases/tissue	Materials	Key research points	References
Preadipocytes	Breast cancer	dECM	Stiff and unfolded fibronectin induced vascular endothelial growth factor expression and led to tumor angiogenesis	Wang et al.^102^
White adipocytes		PEG and Matrigel	ADSC increased TME stiffness through ECM deposition and promoted tumorigenesis	Chandler et al.^103^
ADSC obtained from obese mice		dECM	White adipocytes dedifferentiated on stiffer PEG substrate or compressed Matrigel‐cell constructs by activating the Wnt/β‐catenin signaling pathway	Li et al.^13^
Preadipocytes		PEG/GelMA hybrid hydrogel (200 Pa to 3 kPa)	Adipogenesis was inhibited on high‐stiffness tissue constructs and was further strengthened by cancer cell conditional medium	Yue et al.^12^
ADSC	Bone	Agarose hydrogel	Cyclic hydrostatic pressure could induce the chondrogenic differentiation of porcine ADSC	Carroll et al.^11^
hADSC		Collagen coated plate	2% and 10% strain could inhibit adipogenic differentiation	Huang et al.^99^
hADSC		Gelatin/chitosan scaffold	Compression stress upregulated calcium signaling pathways and SRY‐Box Transcription Factor 9 (Sox‐9) for chondrogenesis	Li et al.^100^

Abbreviations: ADSC, adipose‐derived stem cells; dECM, decellularized extracellular matrix; GelMA; gelatin‐methacryloyl; hADSC, human ADSC; PEG, polyethylene glycol; TME, tumor microenvironment.

The TME varies between obese and lean mice, which is associated with differences in ADSC characteristics. ADSCs obtained from obese mice have more myofibroblasts and produce denser and stiffer ECM than ADSCs from lean mice. Furthermore, decellularized matrices derived from obese ADSC can stimulate mechanosignaling in cancer cells and promote their malignant potential. In contrast, caloric restriction in a mouse model reduced the presence of myofibroblasts in mammary fat and TME stiffness.^101^ ECM derived from tumor‐bearing and obese mammary glands was identified with full‐length collagen VI, contributing to increased breast cancer stiffness and promoting cell invasion by activating Neuron‐Glial Antigen 2 (NG2) ‐ Epidermal Growth Factor Receptor‐EGFR and mitogen‐activated protein kinase signaling. A similar phenomenon was also observed clinically, where collagen VI contents in the TME increased with higher BMI for breast cancer patients.^104^ Cancer‐associated stromal cells can also overexpress the ECM component and modify its conformation to deregulate TME stiffness. Wang et al. used a surface forces apparatus to demonstrate that 3T3‐L1 preadipocytes deposit large amounts of stiff, unfolded fibronectin with altered topology when stimulated by the breast cancer cell‐conditioned medium. This stiffer fibronectin could further decrease 3T3‐L1 adhesion and induce vascular endothelial growth factor expression through the activation of integrin αvβ3, potentially leading to tumor angiogenesis.^102^ Chandler et al. discovered that breast cancer cells can release soluble factors to hinder the adipogenic differentiation of ADSC while simultaneously promoting ADSC proliferation and myofibroblast differentiation. This modification of the ADSC phenotype can result in diverse ECM deposition and contraction, ultimately increasing TME stiffness and promoting tumorigenesis.^103^

Conversely, the mechanical properties of TME significantly influence adipocyte behavior. Yue et al. created a microwell array system using PEG/GelMA hybrid hydrogel with stiffness adjustable in the range of 200 Pa to 3 kPa to investigate the effect of ECM stiffness on the interaction between 3T3‐L1 and breast cancer cells. In high‐stiffness tissue constructs resembling breast cancer, adipogenesis of 3T3‐L1 cells was inhibited, whereas such effects were not observed on low‐stiffness tissue constructs that mimic normal human breast tissue, and these effects were further strengthened by the cancer cell conditional medium.^12^ Li et al. demonstrated that white adipocytes can dedifferentiate under various physical stresses, such as compression and osmotic pressure, by activating the Wnt/β‐catenin signaling pathway. These mechanically induced dedifferentiated adipocytes have a distinctive transcriptome profile and possess the ability of long‐term self‐renewal and serial clonogenicity while not forming teratomas. In addition, mechanically induced dedifferentiated adipocytes could transfer to myofibroblasts within the TME and significantly promote the proliferation of human mammary adenocarcinoma cells both in vitro and in a xenograft model.^13^

Overall, for cancers, cells within adipose tissues establish a harmful cycle that aggravates the disease. They contribute to the deposition of ECM components and adipokines, elevating the stiffness of the TME and fostering the progression, metastasis, and drug resistance of cancer cells. In turn, this process can hinder adipogenic differentiation, trigger adipocyte dedifferentiation, and amplify adipokine secretion, further fueling cancer development. Therefore, therapeutic interventions that focus on disrupting mechanical signaling pathways can have the potential to break this vicious cycle.

### 4.2 Bone diseases

During exercise, the bone matrix is subjected to strains ranging from 2000 to 3500 με. Due to the porous nature of bone, these strains create localized stress concentrations, leading to the generation of pressure gradients and the initiation of local fluid movement in and out of the bone matrix, like squeezing a sponge. Even relatively modest strains in the range of 400 με, which can correspond to seemingly gentle activities like walking, can generate significant fluid flow within the lacunar‐canalicular network in vivo, reaching up to 5 Pa.^105^ ADSC residing on or in proximity to bone surfaces are exposed to this fluid flow resulting from physical exercise. Furthermore, within the bone marrow, minor movements at the interface between marrow and bone, induced by exercise, can create fluid shear independent of strain‐induced fluid flow.^106^ Dynamic shear forces, such as pulsatile fluid flow (PFF),^107^ promote osteogenesis in rat calvarial cells, underscoring their crucial role as a physical factor in mechanotransduction. In addition, the viscosity of red marrow was significantly higher at 400 centipoises (cP) compared to the 40 cP viscosity of fatty marrow.^108^ This discrepancy implies that fluid shear at the interface between bone and marrow, as well as within the marrow itself, can vary significantly due to the fluid dynamic properties of the marrow.^109^ Red and fatty bone marrow can replace each other, and conditions like aging and osteoporosis can increase adipose tissue volume within the marrow while depleting the bone.^110^

**FIGURE 6:**
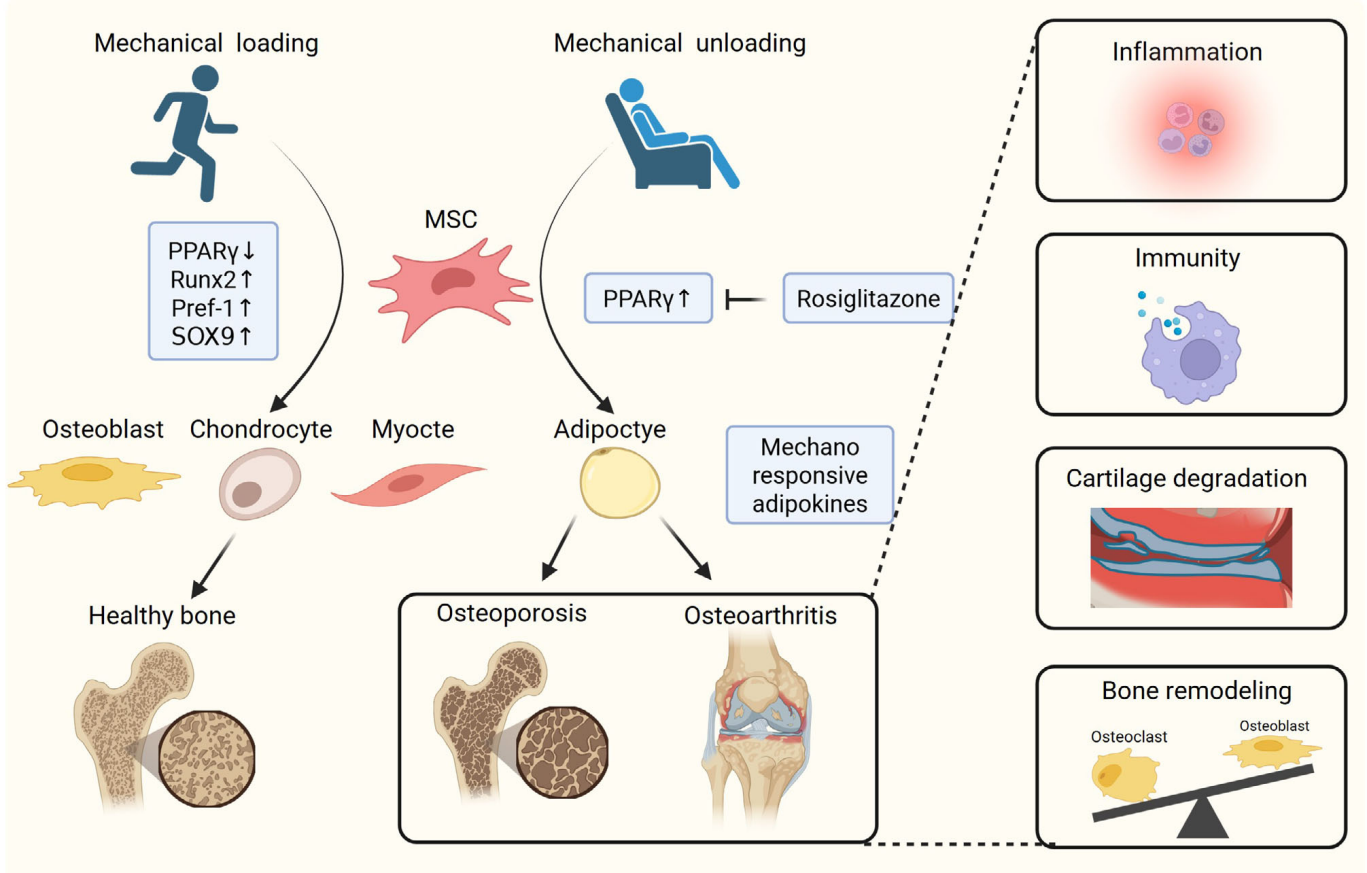
The amount of bone marrow adipose tissue in the bone microenvironment is positively correlated with musculoskeletal disorders, such as osteoarthritis and osteoporosis, while the mechanical loading caused by exercises can inhibit adipogenesis and promote adipose‐derived stem cells osteogenesis. Numerous studies have demonstrated that exercise suppresses marrow adipose tissue formation. Created with BioRender.com. MSC, mesenchymal stem cells; Pref‐1, preadipocyte factor‐1.

Adipocytes within the bone marrow microenvironment are highly mechanosensitive and subject to pronounced modulation by both mechanical unloading and loading conditions.^111^, ^112^, ^113^ Several studies have shown that unloading, like prolonged bedrest and spaceflight, can increase bone marrow adipogenesis^114^ (Figure 6). Prolonged bed rest not only increases bone marrow adiposity but also reduces muscle strength; concurrently, elevated intramuscular lipids and triglycerides contribute to skeletal muscle insulin resistance, which can precipitate postural instability and impair balance and coordination.^115^, ^116^, ^117^ In rat models, both spaceflight‐induced microgravity and terrestrial hindlimb unloading impair bone mineral accrual and promote marrow adipose tissue (MAT) expansion.^118^, ^119^ Conversely, resistive exercise, either alone or combined with low‐magnitude whole‐body vibration, attenuates MAT accumulation.^120^ Mechanistically, mechanical unloading suppresses bone formation markers (runt‐related transcription factor 2 (RUNX2) and osteocalcin) while upregulating adipogenic regulators (PPARγ and C/EBPα) and lipoprotein lipase in the marrow niche.^121^, ^122^ Mechanotransduction at the cellular scale similarly influences mature adipocyte behavior. Pellegrinelli et al. embedded human subcutaneous adipocytes in 3D peptide hydrogels with or without decellularized adipose matrix from obese donors (dMAT) and applied static compression (0%–50%). The results showed that adipocytes demonstrated elevated secretion of proinflammatory cytokines, including IL‐6, IL‐8, and granulocyte colony‐stimulating factor (GCSF), highlighting static strain in bone movement as an anabolic driver of proinflammatory adipocyte phenotype.

Mechanical loading within the bone marrow niche also plays a pivotal role in governing MSC lineage allocation. Murine ADSC were subjected to 3‐day adipogenic induction, followed by exposure to mechanical loading, with a strain of 2000 με and a frequency of 1 Hz using a four‐point bending apparatus. The results showed that mechanical loading significantly reduced the number of cells containing oil droplets and downregulated PPARγ expression while increasing RUNX2 and Pref‐1 expression, highlighting that mechanical strain can impede adipogenic differentiation.^123^ Similar results have been found when human ADSC (hADSC) were cultured on flexible bottomed plates coated with collagen and subjected to mechanical strain using a Flexcell 4000T tensile system at 0.5%, 2%, or 10% strain, with a frequency of 0.5 Hz. At 2% and 10% strain levels, adipogenic differentiation was inhibited.^99^ In contrast, mechanical stress can promote ADSC differentiation to osteogenic, myogenic, and chondrogenic lineages. Ye et al. investigated the osteogenic differentiation of ADSC under uniaxial loading conditions, under a strain of 2400 με and a frequency of 1 Hz. The strain could significantly enhance mineral deposition and the expression of osteogenic‐related genes for hADSC.^124^ PFF shear stress could also influence the osteogenic differentiation of hADSC, where PFF at a frequency of 5 Hz, with an average shear stress of 0.6 Pa, a pulse amplitude of 0.3 Pa, and a peak shear stress of 8.4 Pa/s, were applied to hADSC and induced nitric oxide (NO) production and osteogenic‐related gene expression like osteoblasts.^125^ Li et al. found that the dynamic platen compression with 5% compressive strain at a frequency of 1 Hz could upregulate calcium signaling pathways and Sox‐9, a known inducer of chondrogenesis.^100^ Cyclic hydrostatic pressure could also induce the chondrogenic differentiation of porcine ADSC embedded in agarose scaffolds.^11^

### 4.3 Heart diseases

**FIGURE 7:**
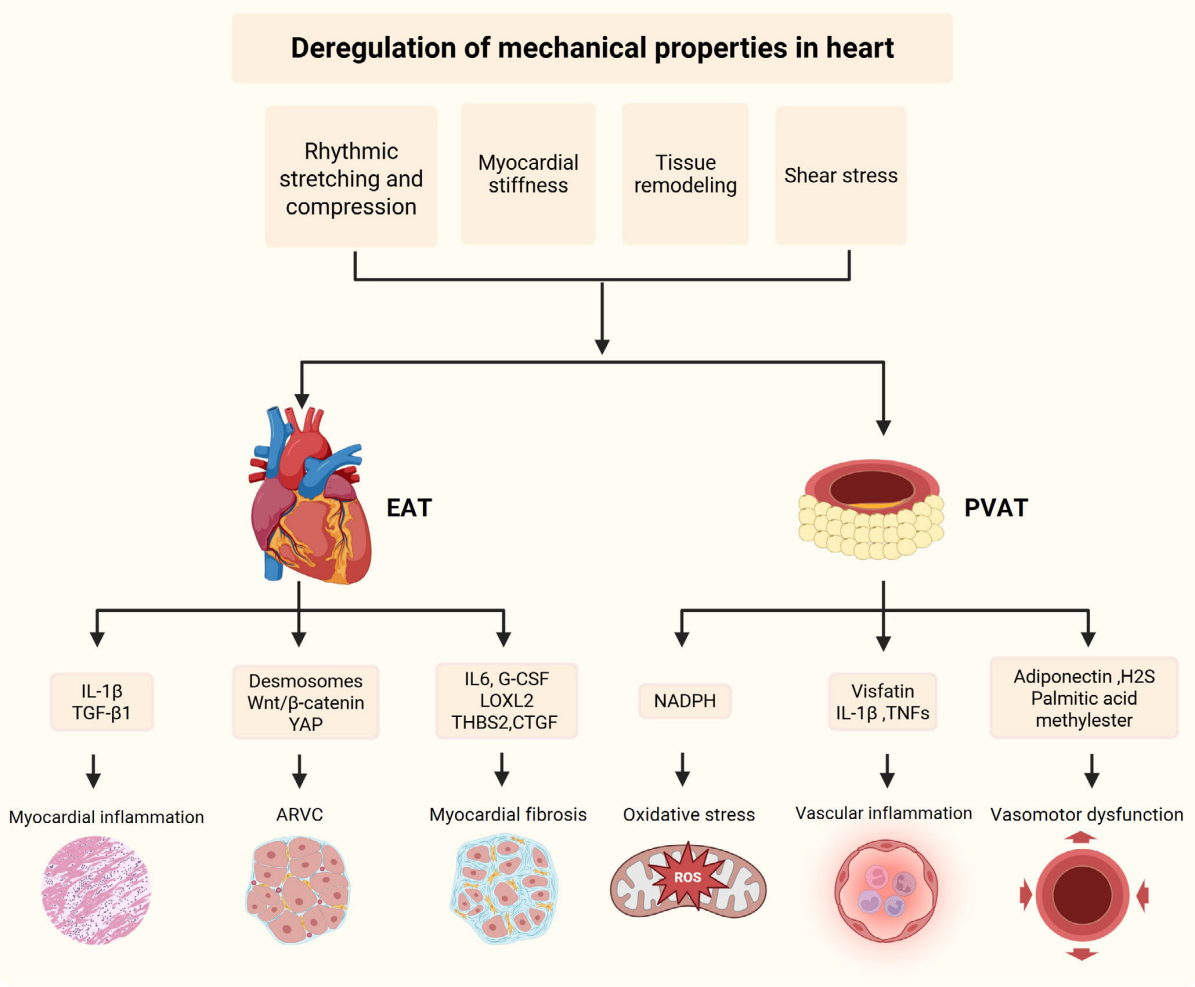
During heart morphogenesis, proper cell allocation and cardiac differentiation are dependent on the mechanical interaction between cells and the extracellular matrix (ECM). Changes in the ECM mechanical properties, such as myocardial fibrosis, induce ectopic lipid accumulation and the enhanced secretion of proinflammatory cytokines. Created with BioRender.com. ARVC, arrhythmogenic right ventricular cardiomyopathy; EAT, epicardial adipose tissue; IL‐6, interleukin 6; PVAT, perivascular adipose tissue; TGF‐β1, transforming growth factor‐β1; TNFs, tumor necrosis factors; YAP, yes‐associated protein; LOXL2, lysyl oxidase Like 2; THBS2, thrombospondin 2; CTGF, connective tissue growth factor; NADPH, nicotinamide adenine dinucleotide phosphate; H2S, hydrogen sulfide.

Cardiac diseases are commonly accompanied by changes in the mechanical microenvironment in the heart (Figure 7). Arrhythmogenic right ventricular cardiomyopathy (ARVC) is a significant form of chronic, progressive, heritable myocardial disorder with a diverse phenotypic range.^126^ Marked by a pronounced susceptibility to sudden cardiac death and advancing heart failure, ARVC phenotypes are exacerbated and incited by intense physical activity. Mechanisms influencing disease penetrance include excessive mechanical stretching, loading, and repetitive adrenergic stimulation.^127^ Approximately half of ARVC patients exhibit mutations in genes responsible for encoding desmosomes. Working alongside integrins and cadherins, these genes contribute to the formation of structural mechanoresponsive elements in the cytoskeleton, such as F‐actin, microtubules, and intermediate filaments. Desmosomes play a crucial role in maintaining the structural integrity of the ventricular myocardium and are implicated in various signal transduction pathways. Mutated desmosomal proteins induce the detachment of cardiac myocytes by disrupting cell adhesions, affecting signaling pathways, and ultimately causing cell death. This process replaces myocardial tissue with fibrofatty adipocytic tissue, weakening the mechanical properties of the heart.^128^ In essence, the mutation or loss of function in cell–cell junctions reduces the mechanical characteristics of cardiomyocytes, a fact substantiated by yes‐associated protein (YAP) inactivation.^129^ Consequently, the ECM in cardiomyocytes emerges as a pliant substrate that influences interstitial cells, adipocytes, and stem cells. In ARVC, mechanical stress prompts alterations in intracellular signaling, including suppressing the Wnt/β‐catenin and YAP signaling pathways. This can potentially trigger the adipogenesis differentiation of progenitor cells in the second heart field.^130^ The diminished mechanical stress may also amplify the adipogenesis process by activating PPARγ and C/EBPα, negatively regulated by Wnt/β‐catenin.^131^

Myocardial fibrosis is a common pathophysiologic feature of many different myocardial conditions,^132^ which refers to the expansion of the cardiac interstitium through the deposition of ECM proteins such as collagens,^133^ along with increased mechanical stress and elevated stiffness. The heightened mechanical stimuli bring about alterations in intracellular biochemical signaling, influencing the behaviors of adipocytes. The rigidity of the ECM plays a crucial role in determining adipogenesis, adipocyte size, and the state of lipid metabolism.^134^ Pathologically, ECM mechanics can affect transcriptional changes in gene expression, leading to the subsequent production of ECM‐related proteins, contributing to adipose tissue fibrosis.^135^ Changes in the ECM hindered adipocytes' capacity to accumulate and release lipids, leading to adipocyte dysfunction, particularly through collagen types I and VI.^136^ Due to the lack of ECM plasticity, adipocytes exhibit characteristics such as ectopic lipid accumulation, reduced lipolysis, the downregulation of insulin‐sensitizing adipokines, and the heightened expression of proinflammatory cytokines and fibrotic mediators.^137^ These factors contribute to conditions such as myocardial disarray and cardiac fibrosis.^138^ In addition, the stiff ECM caused by cardiac fibrosis can trigger the generation of proinflammatory and profibrotic cytokines or adipokines in EAT, intensifying localized inflammation in cardiac fibrosis.^139^ Stimulated adipocytes contribute to the perpetual cycle of inflammation and fibrosis by generating IL‐1β and transforming growth factor (TGF)‐β1, suggesting their significant role in fibrotic remodeling.^140^ Remodeling of the ECM induces mitochondrial dysfunction in adipocytes, while restoring mitochondrial function can inhibit the ability of transplanted ADSC and the release of inflammatory cytokines, ultimately reducing cardiac fibrosis.^141^ A decrease in adipose tissues has been shown to alleviate cardiac fibrosis.^142^

### 4.4 Other diseases

In individuals with obesity, the development of hepatic steatosis is integrally linked to progressive hepatic dysfunction. Although mature adipocytes are not normally present within the liver, persistent nutrient excess induces hepatocytes to accumulate large lipid droplets and acquire adipocyte‐like phenotypes. In animal models and human nonalcoholic fatty liver disease (NAFLD), steatotic hepatocytes upregulate canonical adipogenic markers, including aP2 (FABP4), PPARγ, CD36, as well as proinflammatory cytokines such as TNF‐α, MCP‐1, IL‐6, and IL‐18, which drive both local macrophage activation and systemic insulin resistance.^143^, ^144^, ^145^ At the tissue level, hepatocytes, portal fibroblasts, and activated myofibroblasts serve as the primary cellular sources of ECM in the liver. Hepatic steatosis is accompanied by a marked increase in ECM proteins, including collagen I, collagen IV, laminin, and Alpha‐Smooth Muscle Actin (α‐SMA).^146^, ^147^ Mechanistically, this aberrant ECM accumulation further exacerbates insulin resistance via α1β1 collagen‐binding integrin signaling, which has been shown to regulate hepatocyte insulin sensitivity in response to increased ECM collagen deposition.^148^

Within pancreatic islets, the ECM composition is indispensable for preserving the viability and functional integrity of β‐cells. Following injury or inflammatory stimuli, quiescent pancreatic stellate cells (PSCs) transdifferentiate into activated myofibroblast‐like cells that secrete excessive ECM components, which could increase stromal stiffness, disrupt islet cytoarchitecture, deform ductal networks, and impair endocrine function.^149^ In diabetic islets, particularly around islet blood vessels, ECM accumulation is pronounced.^150^ In db/db mice, enhanced deposition of collagens and laminins in the peri‐islet and exocrine–endocrine interface alters cellular adjacency, suggesting compromised communication between endocrine and exocrine compartments.^151^ Immunohistochemical studies in type 1 diabetic murine models have documented significant HA enrichment within both peri‐islet and intra‐islet ECM compartments, typically colocalized with CD45^+^ immune cell aggregates, highlighting HA's role in modulating local inflammation and islet degradation.^152^

## 5 FUTURE PERSPECTIVES

### 5.1 Biomaterial‐based co‐culture system for adipocytes

Advances in biomaterials as substrates for in vitro cell culture models have greatly enhanced the reliability of co‐culture systems, offering more accurate models to study cell–cell interactions. Our group has previously has developed a co‐culture model for fibroblast and melanoma cells using GelMA as a substrate to investigate of how fibroblasts influence melanoma progression.^153^ Further advancements in co‐culture methods include the use of 3D bioprinting technology, which allows for precise spatial placement of cells to better replicate the architecture of the tissue microenvironment and more closely mimic the cell–cell interactions.^154^ As previously discussed, adipocytes or preadipocytes have been commonly co‐cultured with cancer cells to explore reciprocal interactions. The 3T3‐L1 adipocyte progenitor cells were encapsulated in the hydrogel and fabricated into microwell arrays through micromolding.^12^ Subsequently, the breast cancer cells were seeded in the microwell arrays and observed that cancer cells could inhibit the adipogenesis in high‐stiffness constructs, while the conditioned media from monolayer culture of cancer cells did not show any significant effect, emphasizing the importance of using co‐culture systems.

However, co‐culture models between adipocytes and bone cells or cardiomyocytes are less frequently employed. Research on the bone microenvironment has largely concentrated on the mechanical influences on ADSC differentiation, with less emphasis on direct co‐culture models involving mature bone cells and adipocytes. Improving the co‐culture approaches for bone cells and cardiomyocytes could offer more valuable insights, particularly for a deeper understanding of the influence of adipocytes in disease mechanisms. For example, co‐culturing bone cells and adipocytes within hydrogel substrates under compression allows researchers to examine whether adipocyte dedifferentiation, observed in cancer progression, could similarly occur in the bone microenvironment. Additionally, adipocytes can be 3D bioprinted into hydrogels of varying stiffness and co‐cultured with cardiomyocytes to more closely simulate heart architecture and study the signaling pathways underlying the disease development. Therefore, biomaterial‐based co‐culture systems should be more widely applied to studies involving adipocytes and other cell types. This highlights the need for the development of advanced biomaterials that can more accurately replicate the ECM of specific tissues since ECM provides structural and biochemical cues essential for cell behavior, including adhesion, differentiation, and signaling. Creating biomaterials that closely mimic the native ECM can improve the physiological relevance of in vitro models, allowing researchers to better study cellular interactions and disease mechanisms within a more realistic tissue‐like environment.

### 5.2 Potential combination therapies using biomaterial‐based drug delivery systems

Both mechanotherapeutics and obesity treatments have demonstrated potential efficacy as independent approaches in cancer therapy. Mechanotransduction pathways can be therapeutically targeted by blocking mechanosensors. The suppression of piezo ion channels was currently in the early stages of clinical development, including Gd^3+^, ruthenium red, and GsMTx‐4.^155^ Inhibitors that disrupt the ionic balance of transient receptor potential (TRP) channels by modifying the influx of Ca^2+^ and Na^+^ can trigger apoptosis and impede cancer cell proliferation and migration.^156^ Therapeutic interventions that target adipose tissues in TME are an effective strategy to inhibit cancer progression. Several studies have suggested that patients with T2DM treated with metformin may have a lower cancer incidence than those using other diabetes medications, which can be because metformin activates the AMPK signaling pathway and inhibits insulin‐stimulated growth of breast cancer cell lines.^157^ Metformin also targets cancer stem cells, showing promising results in combination therapy with doxorubicin.^158^ Therefore, combining mechanotherapeutics and adipose tissue treatment could provide potential cancer therapy, which should be further investigated in the future.

Similar phenomena were also observed in the bone diseases. Numerous studies have demonstrated that exercise suppresses BMAT formation, even in cases where BMAT induction is provoked by antidiabetic thiazolidinedione drugs or high‐fat dietary interventions.^159^ This suggests that exercise may protect the structural integrity and the distinctive features of the marrow microenvironment. This was also verified in animal studies, where mice engaged in 6 weeks of daily running displayed increased bone density, enhanced bone quality, and reduced BMAT accumulation regardless of whether they were on a regular or high‐fat diet.^159^ Moreover, the administration of a PPARγ agonist, such as rosiglitazone, enhances stem cell adipogenesis in rodent models and humans,^160^ whereas these effects can be counteracted by dynamic mechanical signals when studied in vitro.^161^ In an in vivo setting, treadmill running in mice treated with rosiglitazone effectively thwarted the shift toward an adipogenic marrow phenotype.^162^ Soft robotics stands out for wearable applications, as its compliant materials afford conformal contact with the human body and offer dynamic compression or massage therapy to enhance lymphatic and blood circulation.^163^ For instance, Zhu et al. developed a wearable soft robotic system that can achieve compressive pressures exceeding 22 kPa at frequencies above the 14 Hz range to deliver dynamic compression and massage therapy.^164^

Early preclinical research and animal models have demonstrated the cardiovascular benefits of several experimental treatment strategies that target adipose tissues. For example, SGLT2 inhibitors have shown direct effects on adipose tissues,^165^ including increased insulin sensitivity and a marked reduction in the amount of EAT in humans,^166^ which can explain the significant cardiovascular benefits among patients with diabetes. Recently developed glucagon‐like peptide‐1 receptor agonists (GLP‐1RAs) that aim for people with diabetes have demonstrated numerous cardiovascular protective effects in individuals. Several cardiovascular outcomes trials (CVOTs) have confirmed the cardiovascular benefits of GLP‐1RAs.^167^, ^168^ These drugs lower plasma lipid levels and blood pressure, both of which contribute to reduced atherosclerosis and decreased CVD risk.^169^ Several pharmacological methods are also used to target the mechanical characteristics of the microenvironment in the heart. In a transverse aortic constriction animal model, suppression of the cross‐linking enzyme LOX homolog 2 has shown a protective effect against the development of diastolic dysfunction by reducing the TGF‐β/Protein Kinase B (PKB) signaling.^170^ YAP (verteporfin),^171^ TGF‐β receptor,^172^ and RHO‐activated kinase inhibitors^173^ have also shown strong antifibrotic properties that reduce or reverse myofibroblast activity and restore ECM mechanical function. Therefore, considering the interaction of adipocytes and the cardiac mechanical microenvironment, the combination of mechanotherapeutics and obesity treatment can provide potential therapy for cardiac diseases.

Biomaterial‐based drug delivery systems have been widely investigated to directly target to disease tissues, control release rates, reduce side effects, and, most importantly, enable combination therapy by co‐delivering multiple drugs and sequentially releasing them, which can act synergistically to maximize treatment outcomes.^174^ Therefore, these drug delivery systems have the potential to be used for the combination of mechanotherapeutics and obesity‐targeted treatments for cancer, bone, and heart diseases. For instance, hollow multi‐shelled nanomaterials,^175^ which feature multiple shell layers separated by cavities and increased surface interfaces, can be used to optimize the sequential delivery of combination therapies. Mechanotherapeutic agents can be loaded onto the outer layers to first weaken physical barriers and disrupt cancer cell growth and migration. Obesity‐targeted drugs can be loaded into the inner layers for delayed release, addressing metabolic factors secreted by adipocytes, such as insulin resistance and adipose‐related signaling, that contribute to tumor progression. This strategic layering enables precise control over the release sequence and enhances therapeutic efficacy by targeting different aspects of tumor development in a coordinated manner.

## 6 CONCLUSIONS

An increasing body of evidence has highlighted the crucial link between mechanical properties in biological tissues and disease. This review summarized the mechanically reciprocal interaction between adipocytes and certain pathologies, including cancers, bone defects, and cardiac conditions. Cells within adipose tissues establish a harmful cycle leading to disease progression through mechanical interaction, where adipocytes deposit ECM components and adipokines to elevate the stiffness of the tissue microenvironment and thus promote disease development, whereas the altered mechanical property can hinder adipogenic differentiation, trigger adipocyte dedifferentiation, amplify inflammatory cytokine secretion, further enhancing disease progression. Therefore, to treat diseases such as cancers, bone defects, and cardiac disorders, the combination of mechanotherapeutics and obesity treatment is a promising way forward.

## AUTHOR CONTRIBUTIONS

**Hangyu Zhou**: Conceptualization and writing‐original draft. **Danni Zhou**: Writing‐original draft. **Miaoben Wu**: Writing‐original draft. **Yuye Huang**: Conceptualization. **Enxing Yu**: Funding acquisition, Writing‐original draft. **Jianing Xie**: Validation. **Yangjian Wang**: Validation. **Shuqin Chen**: Conceptualization. **Qinghua Song**: Supervision. **Kailei Xu**: Funding acquisition, Writing‐review and editing. **Peng Wei**: Conceptualization, Validation, Writing‐review and editing.

## CONFLICT OF INTEREST STATEMENT

The authors declare that they have no known competing financial interests or personal relationships that could have appeared to influence the work reported in this paper.

## Data Availability

All data included in this study is available upon request by contacting the corresponding author.
